# Saúde Cardiovascular e Fibrilação ou Flutter Atrial: Um Estudo Transversal do ELSA-Brasil

**DOI:** 10.36660/abc.20210970

**Published:** 2022-09-15

**Authors:** Itamar S. Santos, Paulo A. Lotufo, Alessandra C. Goulart, Luisa C. C. Brant, Marcelo M Pinto, Alexandre C. Pereira, Sandhi M. Barreto, Antonio L. P. Ribeiro, G Neil Thomas, Gregory Y. H. Lip, Isabela M. Bensenor, NIHR Global Health Research Group on Atrial Fibrillation Management

**Affiliations:** 1 Departamento de Clínica Médica Faculdade de Medicina Universidade de São Paulo São Paulo SP Brasil Departamento de Clínica Médica da Faculdade de Medicina da Universidade de São Paulo , São Paulo , SP – Brasil; 2 Centro de Pesquisa Clínica e Epidemiológica Hospital Universitário Universidade de São Paulo São Paulo SP Brasil Centro de Pesquisa Clínica e Epidemiológica do Hospital Universitário da Universidade de São Paulo , São Paulo , SP – Brasil; 3 Departamento de Clínica Médica Faculdade de Medicina Universidade Federal de Minas Gerais Belo Horizonte MG Brasil Departamento de Clínica Médica da Faculdade de Medicina da Universidade Federal de Minas Gerais , Belo Horizonte , MG – Brasil; 4 Programa de Pós-Graduação em Infectologia e Medicina Tropical Faculdade de Medicina Universidade Federal de Minas Gerais, Belo Horizonte MG Brasil Programa de Pós-Graduação em Infectologia e Medicina Tropical da Faculdade de Medicina da Universidade Federal de Minas Gerais, Belo Horizonte , MG – Brasil; 5 Hospital das Clínicas Faculdade de Medicina Universidade de São Paulo São Paulo SP Brasil Laboratório de Genética e Cardiologia Molecular do Instituto do Coração do Hospital das Clínicas da Faculdade de Medicina da Universidade de São Paulo , São Paulo , SP – Brasil; 6 Departamento de Medicina Preventiva e Social Faculdade de Medicina Universidade Federal de Minas Gerais Belo Horizonte MG Brasil Departamento de Medicina Preventiva e Social da Faculdade de Medicina da Universidade Federal de Minas Gerais , Belo Horizonte , MG – Brasil; 7 Institute of Applied Health Research College of Medical and Dental Sciences University of Birmingham Birmingham Reino Unido Institute of Applied Health Research , College of Medical and Dental Sciences , University of Birmingham , Birmingham – Reino Unido; 8 Chest Hospital and Aalborg Thrombosis Research Unit Department of Clinical Medicine Aalborg University Liverpool Reino Unido Liverpool Centre for Cardiovascular Science, University of Liverpool and Liverpool Heart & Chest Hospital and Aalborg Thrombosis Research Unit , Department of Clinical Medicine , Aalborg University , Liverpool – Reino Unido; * Membros do NIHR Global Health Group para o manejo de fibrilação atrial, listados em ordem alfabética

**Keywords:** Fibrilação Atrial, *Flutter* Atrial, Epidemiologia, Acidente Vascular Cerebral

## Abstract

**Fundamento:**

A associação entre o status de saúde cardiovascular ideal ( *ideal cardiovascular health (* ICVH) e diagnóstico de fibrilação ou *flutter* atrial (FFA) foi menos estudado em comparação a outras doenças cardiovasculares.

**Objetivos:**

Analisar a associação entre o diagnóstico de FFA e métricas e escores de ICVH no Estudo Longitudinal de Saúde do Adulto (ELSA-Brasil).

**Métodos:**

Este estudo analisou dados de 13141 participantes com dados completos. Os traçados eletrocardiográficos foram codificados de acordo com o Sistema de Minnesota, em um centro de leitura centralizado. As métricas do ICVH (dieta, atividade física, índice de massa corporal, tabagismo, glicemia de jeju, e colesterol total) e escores do ICVH foram calculados conforme proposto pela *American Heart Association* . Modelos de regressão logística bruta e ajustada foram construídos para analisar associações de métricas e escores do ICVH com diagnóstico de FFA. O nível de significância foi estabelecido em 0,05.

**Resultados:**

A idade mediana da amostra foi de 55 anos, e 54,4% eram mulheres. Nos modelos ajustados, os escores de ICVH não apresentaram associação significativa com diagnóstico de FFA prevalente [odds ratio (OR):0,96; intervalo de confiança de 95% (IC95%):0,80-1,16; p=0,70). Perfis de pressão arterial ideal (OR:0,33; IC95%:0,1-0,74; p=0,007) e colesterol total ideal (OR:1,88; IC95%:1,19-2,98; p=0,007) foram significativamente associados com o diagnóstico de FFA.

**Conclusões:**

Não foram identificadas associações significativas entre escores de ICVH global e diagnóstico de FFA após ajuste multivariado em nossas análises, devido, ao menos em parte, às associações antagônicas da FFA com métricas de pressão arterial e de colesterol total do ICVH. Nossos resultados sugerem que estimar a prevenção da FFA por meio de escore de ICVH global pode não ser adequado, e as métricas do ICVH devem ser consideradas separadamente.

## Introdução

Em 2010, a *American Heart Association* (AHA) determinou uma meta de redução da mortalidade por doença cardiovascular (DCV) e acidente vascular cerebral (AVC) em 20% em dez anos. ^1^ A principal estratégia para alcançar essa meta incluiu o aumento da prevalência de perfis cardiovasculares ideais, medidos por meio de sete métricas de saúde cardiovascular ideal (ICVH, *ideal cardiovascular health* ): dieta, atividade física, tabagismo, índice de massa corporal (IMC), pressão arterial, glicose plasmática de jejum, e colesterol total. A AHA estabeleceu definições específicas para cada uma dessas métricas, bem como um escore de ICVH para um indivíduo como a soma dos perfis ideais.

A eficácia dessa estratégia está sujeita à força das associações entre cada métrica de ICVH e ocorrência de DCV fatal e não fatal. Alguns autores avaliaram a associação entre escore de ICVH e seus componentes com DCV subclínica, ^2^ DCV clínica ^3^ e mortalidade cardiovascular. ^4^ Uma condição cardiovascular menos estudada neste contexto é a fibrilação ou o *flutter* atrial (FFA). A FFA tem risco de 25% de ocorrência, ^5^ a qual está associada a múltiplos fatores de risco cardiovascular, com uma fração atribuível à população de AVC estimada entre 2 e 6%. ^6 - 8^ Ogunmoroti et al. ^9^ analisaram dados de 6506 participantes do estudo MESA - *Multi-Ethnic Study of Atherosclerosis* e encontraram que indivíduos com um escore ICVH ótimo no período basal apresentaram um risco 27% mais baixo de FFA após um acompanhamento mediano de 11,2 anos em comparação àqueles com escores do ICVH inadequado. Garg et al. ^10^ analisaram dados de 13182 participantes do estudo ARIC ( *Atherosclerosis Risk in Communities* ) e encontraram que, após um seguimento de 25,1 anos, o aumento em cada componente ideal associou-se a um risco 17% menor para FFA. Análises similares foram realizadas por Garg et al. no estudo REGARDS ( *Reasons for Geographic and Racial Differences in Stroke Study* ), ^11^ resultando em uma redução menor (7%) porém significativa no risco para FFA para cada aumento no componente ideal. Recentemente, Lee et al. ^12^ analisaram 208 598 indivíduos que se submeteram a exames de check-up na Coreia do Sul e encontraram, após um seguimento mediano de 7,2 anos, que os escores do ICVH associaram-se significativamente com incidência de FFA.

A maioria das informações sobre a associação entre FFA e métricas e escore do ICVH derivam de grandes coortes dos Estados Unidos; no entanto, estima-se que 72% dos anos de vida ajustados pela incapacidade e 67% das mortes por AVC tenham ocorrido em países com índices sociodemográficos baixos ou médios em 2016. ^13^ A FFA também é comum no Brasil, com frequência de aproximadamente 2% em estudos baseados na atenção primária, ^14^ e frequências maiores associadas ao avanço na idade.

O objetivo do presente estudo foi relatar associações do diagnóstico de FFA com métricas e escore do ICVH no Estudo Longitudinal da Saúde do Adulto (ELSA-Brasil), um estudo brasileiro multicêntrico do tipo coorte de indivíduos com idade entre 35 e 74 anos no momento basal.

## Métodos

O delineamento do estudo ELSA-Brasil ^15^ e o perfil de sua coorte ^16^ foram previamente descritos com detalhes. O ELSA-Brasil é um estudo multicêntrico tipo coorte em andamento no Brasil, em que foram incluídos 15105 servidores públicos ativos ou aposentados, com idade entre 35 e 74 anos, de seis instituições de seis cidades brasileiras. A avaliação basal ocorreu entre agosto de 2008 e dezembro de 2010. Nesse período, uma equipe treinada conduziu entrevistas presenciais, e exames clínicos, laboratoriais e de imagem. Após o período basal, todos os participantes eram contatados por telefone anualmente. De agosto de 2012 a dezembro de 2014, todos os participantes foram convidados para uma segunda visita, em que foram aplicados novos questionários, juntamente com avaliações clínicas e laboratoriais. Um total de 14014 (92,8%) indivíduos participaram dessa reavaliação. No presente artigo, as informações usadas foram obtidas dessa segunda avaliação presencial, salvo se indicado o contrário. O protocolo do estudo esteve de acordo com as normas éticas da Declaração de Helsinki de 1975 e foi aprovado pelo comitê de ética de todas as instituições participantes do ELSA-Brasil. Um termo de consentimento de todos os participantes foi obtido.

### Amostra do estudo

Dos 14014 indivíduos que estiveram presentes na segunda avaliação presencial, 505 foram excluídos por falta de informação de eletrocardiograma (ECG) e 368 participantes por métricas do ICVH faltantes. Assim, nossa amostra principal consistiu em 13141 indivíduos. Para algumas análises de sensibilidade, a amostra restringiu-se a 12307 indivíduos sem relato de infarto do miocárdio, AVC ou insuficiência cardíaca prévio.

### Coleta de dados

Os protocolos do ELSA-Brasil ^17 , 18^ para a avaliação de dados antropométricos, clínicos e laboratoriais não mudaram entre a primeira e a segunda avaliação. Idade, sexo, raça, nível de escolaridade, e renda familiar mensal foram autorrelatadas e estratificadas. A renda mensal foi analisada como múltiplos de salários mínimos brasileiros (a média de um salário mínimo brasileiro durante a coleta de dados foi de aproximadamente 310 dólares americanos). Medidas de pressão arterial sistólica (PAS) e pressão arterial diastólica (PAD) foram aferidas utilizando-se um aparelho oscilométrico (Omron HEM 705 CPINT). Três medidas foram tomadas em intervalos de um minuto após um período de descanso de cinco minutos com os indivíduos sentados. A média da segunda e da terceira medida foi considerada para definição da PAS e PAD. A glicemia de jejum foi determinada pelo método da hexoquinase e o colesterol total usando o método do colesterol oxidase, realizados em um equipamento ADVIA 1200 Siemens®.

### Diagnóstico de FFA

Os registros de ECG foram obtidos no ELSA-Brasil conforme descrito anteriormente. ^19 , 20^ O ECG nas avaliações presenciais foi realizado utilizando um aparelho Burdick Atria 6100, calibrado a 10mm/mV e velocidade de 25 mm/segundo. Os registros foram transmitidos para o centro de leitura no Centro de Investigação em Minas Gerais. Os traçados foram analisados seguindo-se o sistema de Glasgow e codificados de acordo com o Sistema de Minnesota. Os códigos selecionados (incluindo FFA) foram manualmente revisados por uma equipe treinada. Esses métodos foram adotados em todas as avaliações do ELSA-Brasil até o presente. O diagnóstico de FFA neste artigo foi definido como sua presença no traçado de ECG no período basal ou na segunda visita.

### Escore ICVH

Informações detalhadas sobre o sistema de escore do ICVH no ELSA-Brasil podem ser encontradas em outro estudo. ^21^ As seguintes definições foram usadas para as sete métricas do ICVH, baseadas nas recomendações da AHA: ^1^

1. Dieta: quatro componentes adequados de (a) ≥ 4 porções de frutas e verduras por dia, (b) ≥200 g de peixe por semana, (c) ≥ 2 porções de grãos integrais ricos em fibra por dia, (d) ≤ 450 kcal de bebidas adoçadas com açúcar por semana, e (e) consumo de sódio ≤1500 mg/dia;
2. Atividade física: ≥ 75 minutos / semana ou atividade física rigorosa, ou ≥ 150 minutos / semana de atividade física moderada, ou ≥ 150 minutos / semana de atividade física moderada e atividade física vigorosa;
3. Tabagismo: nunca ter fumado ou ter idade ao parar de fumar ao menos dois anos menor que a idade atual;
4. IMC < 25 kg/m ^2^ ;
5. Pressão arterial: PAS < 120 mmHg e PAD < 80 mmHg, sem medicação anti-hipertensiva;
6. Glicemia de jejum: < 100mg/dL sem uso de medicamentos hipoglicemiantes;
7. Colesterol total < 200 mg/dL, sem uso de medicamentos hipolipemiantes.

Todas as informações para o escore ICVH foram obtidas da segunda visita, exceto informações relacionadas à dieta. A métrica do ICVH correspondente à dieta foi avaliada a partir de informações obtidas do questionário de frequência alimentar, aplicado no momento basal do ELSA-Brasil. O escore ICVH global foi calculado como a soma de todos os perfis ideais (intervalo: 0 a 7 pontos) Escores do estilo de vida dos participantes (dieta, atividade física, tabagismo, e IMC, intervalo; 0-4) e saúde (pressão arterial, glicemia de jejum, colesterol total; intervalo 0-3) foram avaliados separadamente.

### Análise estatística

As variáveis categóricas foram apresentadas em contagens e proporções, e as variáveis contínuas em medianas e intervalos interquartis. A normalidade dos dados foi avaliada usando gráficos de densidade e o teste de Anderson-Darling. Nós calculamos a sensibilidade e a especificidade de diferentes pontos de corte do escore do ICVH global para classificar indivíduos com ou sem FFA na amostra. Construímos uma curva característica de operação do receptor (ROC) usando esses valores e calculamos a área sob a curva ROC para estimar a capacidade discriminatória dos escores do ICVH para identificar os indivíduos com FFA neste cenário. Modelos de regressão logística binária foram construídos para analisar a associação de métricas e escores do ICVH (global, estilo de vida, e saúde) com o diagnóstico de FFA. Esses modelos foram apresentados na forma bruta ou ajustada quanto à idade, sexo e raça. Foram feitas análises de sensibilidade restringindo a amostra a indivíduos sem relato de história de infarto do miocárdio, AVC ou insuficiência cardíaca. Após observar uma associação paradoxal entre a métrica doo ICVH de colesterol total e FFA, realizamos algumas análises *a posteriori* , (1) excluindo indivíduos em uso de estatina, uma vez que alguns autores indicaram um benefício potencial das estatinas sobre incidência de fibrilação atrial; ^22 , 23^ (2) excluindo a métrica de colesterol total do escore de saúde e do escore global do ICVH. Os modelos de regressão logística binária bruta e ajustada também foram apresentados para analisar a associação entre esses escores modificados e o diagnóstico de FFA. As análises foram realizadas usando o programa R, versão 4.0.0. O nível de significância foi estabelecido em 5%.

## Resultados

A Tabela 1 resume as características da amostra. A idade mediana foi de 55 anos, e 7147 (54.4%) eram mulheres. A maioria dos participantes era da raça branca, tinham educação superior e renda familiar entre seis e 15 salários mínimos brasileiros (aproximadamente 1860 a 4650 dólares americanos/mês). A FFA foi detectada em traçados de ECG em 80 (0,6%) participantes. A métrica do ICVH mais frequente foi tabagismo, presente em 11548 (87,9%) participantes. A Tabela 1 Suplementar apresenta as características dos participantes do estudo após exclusão daqueles com história de infarto do miocárdio, AVC ou insuficiência cardíaca.

**Tabela 1:** Características da amostra do estudo

	Sem fibrilação ou *flutter* atrial (N=13061)	Com Sem fibrilação ou *flutter* atrial (N=80)	Total (N=13141)
**Idade (anos; média ± DP)**	55,0 [49,0 - 62,0]	64,5 [57,0 - 73,2]	55,0 [49,0 - 62,0]
**Sexo feminino (N (%))**	7117 (54,5%)	30 (37,5%)	7147 (54,4%)
**Raça (N (%))**			
Branca	6750 (52,3%)	46 (60,5%)	6796 (52,3%)
Mulata	3625 (28,1%)	21 (27,6%)	3646 (28,1%)
Negra	2071 (16,0%)	9 (11,8%)	2080 (16,0%)
Outras	471 (3,6%)	0 (0,0%)	471 (3,6%)
**Nível educacional, N (%)**			
Ensino médio incompleto	1472 (11,3%)	13 (16,2%)	1485 (11,3%)
Ensino médio completo	4110 (31,5%)	25 (31,2%)	4135 (31,5%)
Superior	7476 (57,3%)	42 (52,5%)	7518 (57,2%)
**Renda familiar mensal (N (%))**			
< 6 SMs	2816 (21,7%)	16 (20,0%)	2832 (21,6%)
6 - 15 SMs	6112 (47,0%)	29 (36,2%)	6141 (46,9%)
> 15 SMs	4073 (31,3%)	35 (43,8%)	4108 (31,4%)
**Uso de medicamento anti-hipertensivo (N (%))**	5800 (44,4%)	61 (76,2%)	5861 (44,6%)
**Uso de medicamento hipoglicemiante, N (%)**	2577 (20,9%)	30 (39,5%)	2607 (21,0%)
**Uso de medicamento hipolipemiante, N (%)**	6658 (51,0%)	37 (46,2%)	6695 (51,0%)
**Métricas do ICVH (N (%))**			
Dieta	177 (1,4%)	0 (0,0%)	177 (1,3%)
Atividade física	3488 (26,7%)	15 (18,8%)	3503 (26,7%)
Índice de massa corporal	4229 (32,4%)	27 (33,8%)	4256 (32,4%)
Tabagismo	11474 (87,8%)	74 (92,5%)	11548 (87,9%)
Pressão arterial	4499 (34,4%)	7 (8,8%)	4506 (34,3%)
Glicemia de jejum	5927 (45,4%)	23 (28,7%)	5950 (45,3%)
Colesterol Total	5213 (39,9%)	38 (47,5%)	5251 (40,0%)
**Escore ICVH (média ± DP)**			
Global	3,0 [2,0 - 4,0]	2,0 [1,0 - 3,0]	3,0 [2,0 - 4,0]
Estilo de vida	1,0 [1,0 - 2,0]	1,0 [1,0 - 2,0]	1,0 [1,0 - 2,0]
Saúde	1,0 [0,0 - 2,0]	1,0 [0,0 - 1,0]	1,0 [0,0 - 2,0]

*SM: salário mínimo; o salário mínimo brasileiro durante a coleta de dados foi de aproximadamente 310 dólares americanos. ICVH: Ideal cardiovascular health (saúde cardiovascular ideal). Fonte: os autores.*

A Figura Suplementar 1 apresenta a curva ROC referente à acurácia dos escores de ICVH global em identificar indivíduos com FFA na amostra. A área sob a curva ROC é 0,59, indicando um poder discriminatório relativamente baixo dos escores de ICVH global neste cenário. Mais detalhes são apresentados na Tabela Suplementar 2 , que mostra valores de sensibilidade e especificidade para diferentes pontos de corte do escore de ICVH global.

A Tabela 2 apresenta os valores de *odds ratio* (OR) e respectivos intervalos de confiança (IC95%) para a associação de escores e métricas de ICVH com prevalência de FFA. Em toda a amostra, os escores de ICVH foram inversamente associados com o diagnóstico de FFA no modelo bruto (p<0,001) e no modelo ajustado (p=0,007). A exclusão dos indivíduos em uso de estatina das análises levou a uma perda de significância da associação entre o perfil de colesterol total no ICVH e a FFA nos modelos ajustados (OR: 2,22; IC95%: 0,86 – 5,70; p=0,098). Contudo, essa perda de significância parece ser devida principalmente a uma perda de poder estatístico, uma vez que 46,2% dos participantes com FFA e 51,0% dos participantes sem FFA usavam estatinas.

**Tabela 2:** Odds ratios (IC95%) para a associação de escores e métricas de ICVH e diagnóstico de fibrilação ou *flutter* atrial

	Toda a amostra		Excluindo pacientes que relataram história de infarto do miocárdio, acidente vascular cerebral ou insuficiência cardíaca	

	Bruta	Ajustada para idade, sexo e raça	Bruta	Ajustada para idade, sexo e raça
**Escore de ICVH global**	0,80 (0,67 - 0,95)*	0,96 (0,80 - 1,16)	0,87 (0,71 - 1,08)	1,01 (0,81 - 1,26)
**Escore de estilo de vida do ICVH**	0,95 (0,71 - 1,26)	0,96 (0,70 - 1,30)	1,00 (0,70 - 1,42)	0,96 (0,67 - 1,38)
Dieta	^†^	^†^	^†^	^†^
Atividade física	0,63 (0,36 - 1,11)	0,65 (0,37 - 1,15)	0,71 (0,36 - 1,38)	0,67 (0,34 - 1,31)
Índice de massa corporal	1,06 (0,67 - 1,69)	1,19 (0,74 - 1,92)	1,33 (0,77 - 2,32)	1,38 (0,79 - 2,40)
Tabagismo	1,71 (0,74 - 3,93)	1,40 (0,60 - 3,23)	1,08 (0,46 - 2,52)	0,92 (0,39 - 2,18)
**Escore de saúde do ICVH**	0,66 (0,51 - 0,85)*	0,96 (0,73 - 1,26)	0,77 (0,57 - 1,03)	1,06 (0,77 - 1,46)
Pressão arterial	0,18 (0,08 - 0,40)*	0,33 (0,15 - 0,74)*	0,23 (0,10 - 0,53)*	0,36 (0,15 - 0,87)*
Glicose	0,49 (0,30 - 0,79)*	0,79 (0,48 - 1,31)	0,65 (0,37 - 1,13)	0,97 (0,54 - 1,74)
Colesterol Total	1,36 (0,88 - 2,12)	1,88 (1,19 - 2,98)*	1,62 (0,95 - 2,79)	2,14 (1,23 - 3,72)*

ICVH: Ideal cardiovascular health (saúde cardiovascular ideal); † Estimativas de odds ratio para a associação entre métrica de dieta do ICVH e fibrilação ou flutter atrial não puderam ser estimadas devido à ausência de indivíduos com um perfil ótimo de dieta e um diagnóstico de fibrilação ou flutter atrial; * p<0,05. Fonte: os autores

Uma vez que as associações da FFA com a pressão arterial e a métrica de colesterol total do ICVH eram antagônicas, a FFA não se associou com escore de ICHV global nos modelos ajustados (p=0.76). Após exclusão de indivíduos com relato de infarto do miocárdio ou insuficiência cardíaca prévia, as associações da FFA com pressão arterial (p=0.023) e métrica de colesterol total do ICVH (p=0,007) nos modelos ajustados foram mantidas.

Também verificamos se os escores de saúde e os escores de ICVH global foram associados com FFA após exclusão da métrica de colesterol total. O escore de ICVH global mostrou-se inversamente associado com um diagnóstico de FFA nos modelos brutos (OR: 0,70; IC95%: 0,57 – 0,86; p=0,001) com uma tendência não significativa a uma associação inversa nos modelos ajustados (OR: 0,84; IC95%: 0,67 – 1,04; p=0,11). Os escores de saúde do ICVH foram inversamente associados com um diagnóstico de FFA no modelo absoluto (OR: 0,41; IC95%: 0,28 – 0,59; p<0,001) e no modelo ajustado (OR: 0,63; IC95%: 0,42 – 0,93; p=0,020).

## Discussão

Na amostra do ELSA-Brasil, os escores de ICVH global foram associados com prevalência de FFA nos modelos brutos. No entanto, os escores foram ajustados com valores de área sob a curva ROC e a significância estatística desapareceu nos modelos ajustados para idade, sexo e raça. Analisando as métricas de ICVH separadamente, encontrou-se uma forte associação inversa entre a métrica de pressão arterial do ICVH e a FFA. Uma associação positiva, mas paradoxal, foi observada entre métrica de colesterol total do ICVH e FFA: uma métrica de colesterol total ideal do ICVH foi associada com diagnóstico de FFA. A restrição da amostra para indivíduos sem relato de infarto do miocárdio, AVC ou insuficiência cardíaca levou a resultados similares. A exclusão da métrica do colesterol total do ICVH levou a uma associação inversa significativa entre escores de saúde do ICVH e FFA.

A importância da hipertensão como um fator de risco para FFA está bem definida na literatura. Em uma revisão sistemática de fatores de risco para FFA Allan et al. ^24^ revisaram dados de 32 estudos de coortes de base populacional, incluindo mais de 20 milhões de indivíduos. Os autores encontraram que o diagnóstico de hipertensão e pressão arterial sistólica elevada foram consistentemente associados com incidência de FFA. Recentemente, Rattani et al. ^25^ utilizaram dados do estudo ARIC para calcular o risco atribuível na população de hipertensão para FFA. Os autores definiram hipertensão de acordo com os critérios do Sétimo Relatório do *Joint National Committee* (≥140/90 mmHg) e as diretrizes da *American Heart Association/American College of Cardiology* de 2017 (≥130/80 mmHg), e encontraram valores de fração atribuível populacional de 11% e 13%, respectivamente. Como esperado, a presença de um perfil ideal de pressão arterial também foi inversamente associada à FFA nos estudos de tipo coorte ARIC, ^10^ REGARDS, ^11^ e MESA. ^9^

Embora o colesterol total (principalmente por fração LDL-colesterol) seja um fator de risco importante para DCV aterosclerótica, sua associação com FFA não é tão bem estabelecida. Outros autores indicaram o paradoxo do colesterol na FFA, descrevendo níveis mais baixos de colesterol em indivíduos com FFA, em comparação a controles em vários cenários, tais como indivíduos tratados em centros de atenção primária, ^26 , 27^ ou mesmo indivíduos de amostras da comunidade. ^28^ Em uma revisão sistemática recente, Guan et al. ^29^ encontraram que níveis elevados de colesterol total (definidos em pontos de corte entre 220 e 260 mg/dL, ou com base na distribuição empírica na amostra) foram associados a uma razão de risco agrupado ( *pooled hazard ratio* ) para FFA de 0,81 (IC95%: 0,72-0,92), o que foi consistente como nossos achados. Na mesma revisão sistemática, análises utilizando níveis de LDL-colesterol em vez de níveis de colesterol total mostraram resultados similares.

Existem evidências de que as estatinas possam tem potencial benefício sobre a incidência de fibrilação atrial. ^22 , 23^ No entanto, a métrica do ICVH considera um perfil como não ideal se os indivíduos estão em uso de medicamentos hipolipemiantes, independentemente de seus níveis de colesterol total. Portanto, poderia ser argumentado que a associação paradoxal entre perfil de ICVH quanto ao colesterol total e FFA é explicada pelo uso de estatina; porém, nossos dados sugerem que esse não é o caso. A proporção de indivíduos de nossa amostra usando medicamentos hipolipemiantes é similar. Ainda, embora a exclusão de indivíduos que usam estatinas tenha levado à perda de significância estatística, isso provavelmente tenha ocorrido pelo elevado número de exclusões dessa análise de sensibilidade (51,0% da nossa amostra), uma vez que as estimativas do OR também eram similares (1,88 para toda a amostra; 2,22 após exclusão dos pacientes usando agentes hipolipemiantes).

O critério usado como a métrica de colesterol total do ICVH (<200 mg/dL sem uso de hipolepimantes) é mais rígido que aqueles adotados nos estudos incluídos nesta revisão sistemática. Os estudos coorte ^9 - 11^ que avaliaram os escores de ICVH e sua relação com diagnóstico de FFA também investigaram a métrica de colesterol total do ICVH separadamente. Em todos os casos, as estimativas do risco sugeriram maior chance para FFA nos indivíduos com colesterol total ideal, mas sem diferença estatisticamente significativa. Essa ausência de significância nesses estudos contrasta com nossos achados de uma associação significativa, positiva, mas paradoxal entre a métrica de colesterol total ICVH e o diagnóstico de FFA nos modelos ajustados. Essa associação estatisticamente significativa em nosso estudo foi evidente mesmo com menos casos de FFA na amostra, em comparação a publicações anteriores. Diferenças de populações e delineamento dos estudos, podem ser parcialmente responsáveis por tal discrepância. Similar ao que se observa nos Estados Unidos, indivíduos brasileiros da raça negra apresentam uma prevalência maior de hipertensão ^30^ e uma prevalência mais baixa de dislipidemia ^31^ em comparação aos de raça branca. Dada a forte associação inversa entre pressão arterial e FFA, poderia ser argumentado que a raça seria um fator de confusão na associação positiva entre perfil de colesterol total do ICVH e diagnóstico de FFA. No entanto, é importante notar que nossos resultados se mantiveram mesmo após o ajuste por raça. Análises longitudinais futuras do ELSA-Brasil, incluindo a ocorrência de mais casos de FFA, irá esclarecer se o paradoxo do colesterol em indivíduos com FFA é particularmente forte na população brasileira.

Em 2018, Isakadze et al. ^32^ propuseram que o uso do escore ICVH para melhorar a saúde cardiovascular global pode reduzir substancialmente a prevalência de FFA. Embora evidências atuais sugiram que tal fato seja verdadeiro, nossos resultados e dados prévios também destacam que métricas individuais do ICVH podem ter efeitos diferentes (e mesmo antagônicos) sobre a prevalência da FFA. Assim, é discutível se o progresso na prevenção da FFA resultante não só de melhorias nos escores do ICVH devam ser estimadas considerando não só o impacto de todas as métricas do ICVH em conjunto como também cada métrica separadamente.

O presente estudo tem pontos fortes. Primeiro, o estudo avaliou dados de uma grande amostra no Brasil. Em nosso conhecimento, este é o primeiro estudo a analisar a associação entre FFA e métricas do ICVH em uma amostra da América do Sul com diferentes características comparadas às amostras analisadas em estudos prévios. Os traçados de ECG também foram analisados em um local de leitura centralizado, usando um protocolo padronizado, ^19^ e os critérios de escore do ICVH pôde ser aplicado com mínimas adaptações.

O estudo também precisa ser interpretado no contexto de suas limitações. A amostra deste estudo incluiu um pequeno número de participantes com FFA. Isso deve-se muito provavelmente à alta proporção de indivíduos com idade inferior a 60 anos de idade. Com o passar do tempo, os casos incidentes de FFA podem aumentar o poder de nossas análises. Isso pôde ter influenciado nosso resultado de não associação entre diagnóstico de FFA e escores de ICVH. O questionário de frequência alimentar não foi aplicado na segunda visita dos participantes no estudo ELSA-Brasil; portanto, considerou-se que os participantes mantiveram o status de dieta no ICVH após o basal. A prevalência de uma dieta ideal (segundo critérios do ICVH) foi muito baixa no período basal do ELSA-Brasil (1,3%), ^21^ que é consistente com os achados de outras populações. ^33^ Assim, é improvável que uma grande proporção de participantes teria adotado uma dieta ideal entre o período basal e a segunda visita. A FFA foi definida com base somente nos traçados de ECG do ELSA-Brasil e, apesar de conferir alta especificidade, FFA paroxística pode ser pouco representada em nossas análises.

## Conclusões

Não foram observadas associações significativas entre escores de ICVH global e diagnóstico de FFA em nossas análises, devido, ao menos em parte, às associações antagônicas de FFA com pressão arterial e colesterol total. Nossos resultados sugerem que estimar o efeito da prevenção de FFA usando escores de ICVH global pode não ser adequado, e as métricas do ICVH devem ser consideradas separadamente.

## * Material suplementar

Para informação adicional da Figura Suplementar 1, por favor, clique aqui .

Para informação adicional das Tabelas Suplementares, por favor, clique aqui .
