# Supplementary material for: Saúde Cardiovascular e Fibrilação ou Flutter Atrial: Um Estudo Transversal do ELSA-Brasil
Source: Arq Bras Cardiol. 2022 Sep 15;119(5):724–31. [Article in Portuguese] doi: 10.36660/abc.20210970 (PMC9750207; doi:10.36660/abc.20210970)
Supplement: Translation

# Translation: Cardiovascular Health and Atrial Fibrillation or Flutter: A Cross-Sectional Study from ELSA-Brasil

**Authors:** Itamar S. Santos, Paulo A. Lotufo, Alessandra C. Goulart, Luisa C. C. Brant, Marcelo M Pinto, Alexandre C. Pereira, Sandhi M. Barreto, Antonio L. P. Ribeiro, G Neil Thomas, Gregory Y. H. Lip, Isabela M. Bensenor, NIHR Global Health Research Group on Atrial Fibrillation Management

**Affiliations:** 1 Brazil Departamento de Clínica Médica da Faculdade de Medicina da Universidade de São Paulo, São Paulo, SP – Brazil; 2 Brazil Centro de Pesquisa Clínica e Epidemiológica do Hospital Universitário da Universidade de São Paulo, São Paulo, SP – Brazil; 3 Brazil Departamento de Clínica Médica da Faculdade de Medicina da Universidade Federal de Minas Gerais, Belo Horizonte, MG – Brazil; 4 Brazil Programa de Pós-Graduação em Infectologia e Medicina Tropical da Faculdade de Medicina da Universidade Federal de Minas Gerais, Belo Horizonte, MG – Brazil; 5 Brazil Laboratório de Genética e Cardiologia Molecular do Instituto do Coração do Hospital das Clínicas da Faculdade de Medicina da Universidade de São Paulo, São Paulo, SP – Brazil; 6 Brazil Departamento de Medicina Preventiva e Social da Faculdade de Medicina da Universidade Federal de Minas Gerais, Belo Horizonte, MG – Brazil; 7 UK Institute of Applied Health Research, College of Medical and Dental Sciences, University of Birmingham, Birmingham – UK; 8 UK Liverpool Centre for Cardiovascular Science, University of Liverpool and Liverpool Heart & Chest Hospital and Aalborg Thrombosis Research Unit, Department of Clinical Medicine, Aalborg University, Liverpool – UK; * Members of the NIHR Global Health Group on Atrial Fibrillation Management listed in alphabetical order

**Keywords:** Atrial Fibrillation, Atrial Flutter, Epidemiology, Stroke

## Abstract

**Background:**

The association between ideal cardiovascular health (ICVH) status and atrial fibrillation or flutter (AFF) diagnosis has been less studied compared to other cardiovascular diseases.

**Objective:**

To analyze the association between AFF diagnosis and ICVH metrics and scores in the Brazilian Longitudinal Study of Adult Health (ELSA-Brasil).

**Methods:**

This study analyzed data from 13,141 participants with complete data. Electrocardiographic tracings were coded according to the Minnesota Coding System, in a centralized reading center. ICVH metrics (diet, physical activity, body mass index, smoking, blood pressure, fasting plasma glucose, and total cholesterol) and scores were calculated as proposed by the American Heart Association. Crude and adjusted binary logistic regression models were built to analyze the association of ICVH metrics and scores with AFF diagnosis. Significance level was set at 0.05.

**Results:**

The sample had a median age of 55 years and 54.4% were women. In adjusted models, ICVH scores were not significantly associated with prevalent AFF diagnosis (odds ratio [OR]:0.96; 95% confidence interval [95% CI]:0.80-1.16; p=0.70). Ideal blood pressure (OR:0.33; 95% CI:0.15–0.74; p=0.007) and total cholesterol (OR:1.88; 95% CI:1.19–2.98; p=0.007) profiles were significantly associated with AFF diagnosis.

**Conclusions:**

No significant associations were identified between global ICVH scores and AFF diagnosis after multivariable adjustment in our analyses, at least partially due to the antagonistic associations of AFF with blood pressure and total cholesterol ICVH metrics. Our results suggest that estimating the prevention of AFF burden using global ICVH scores may not be adequate, and ICVH metrics should be considered in separate.

## Introduction

In 2010, the American Heart Association (AHA) set a decade-long goal to decrease cardiovascular disease (CVD) and stroke mortality by 20%.^1^ The main strategy to achieve this goal included raising the prevalence of ideal cardiovascular profiles, measured by seven ideal cardiovascular health (ICVH) metrics: diet, physical activity, smoking, body mass index (BMI), blood pressure, fasting plasma glucose, and total cholesterol. The AHA established specific definitions for each of these metrics, as well as a ICVH score for an individual as the sum of the ideal profiles.

The effectiveness of such a strategy is subject to the strength of the associations between each ICVH metric and occurrence of fatal or non-fatal CVD. Some authors evaluated the association of the ICVH score and its components with subclinical CVD,^2^ clinical CVD^3^ and cardiovascular mortality.^4^ A less studied cardiovascular condition in this context is atrial fibrillation or flutter (AFF). AFF has a lifetime risk of 25%,^5^ is associated with multiple cardiovascular risk factors, and is a major risk factor for stroke, with population attributable fractions of stroke estimated between 2% and 6%.^6 - 8^ Ogunmoroti et al.^9^ analyzed data from 6,506 participants from the Multi-Ethnic Study of Atherosclerosis (MESA) and found that individuals with an optimum ICVH score at the baseline had a 27% lower risk of AFF after a median follow-up of 11.2 years as compared to those with inadequate ICVH scores. Garg et al.^10^ analyzed data from 13,182 Atherosclerosis Risk in Communities (ARIC) participants and found that, after a follow-up of 25.1 years, each ideal component increase was associated with a 17% lower risk for AFF. Similar analyses were also performed by Garg et al. in the Reasons for Geographic and Racial Differences in Stroke Study (REGARDS),^11^ resulting in a smaller (7%) although significant reduction in the risk for AFF for each ideal component increase. Recently, Lee et al.^12^ analyzed 208,598 individuals who underwent national health check-up examinations in South Korea and found, after a median follow-up of 7.2 years, that ICVH scores were significantly associated with incident AFF.

Most information about the association between AFF and ICVH metrics and score come from large cohorts in the United States; however, 72% of global disability-adjusted life-years and 67% of deaths due to stroke were estimated to occur in countries with middle or low sociodemographic index in 2016.^13^ AFF is also common in Brazil, with a frequency of about 2% in primary care-based studies,^14^ with higher frequencies associated with advancing age.

The aim of the present study is to report the associations between AFF diagnosis and ICVH metrics and score in the Brazilian Longitudinal Study of Adult Health (ELSA-Brasil), a Brazilian multicenter cohort study of individuals of 35-74 years of age at the baseline.

## Methods

The ELSA-Brasil design^15^ and cohort profile^16^ are detailed elsewhere. ELSA-Brasil is an ongoing multicenter cohort study in Brazil which enrolled 15,105 active or retired civil servants, from 35 to 74 years of age at baseline, from six institutions in six Brazilian cities. Baseline assessment occurred between August 2008 and December 2010. During this period, trained staff conducted in-person interviews, as well as clinical, laboratory, and imaging exams. After the baseline, all participants received a yearly telephone follow-up contact. From August 2012 to December 2014, all participants were invited to a second visit, during new questionnaires were administered, together with clinical and laboratory evaluations. A total of 14,014 (92.8%) individuals were reassessed. In the present article, information was obtained from the second visit, unless stated otherwise. The study protocol conformed to the ethical guidelines of the 1975 Declaration of Helsinki and was approved by the Institutional Review Boards of all institutions where ELSA-Brasil investigation centers are located. Informed consent was obtained from each participant.

### Study sample

From 14,014 individuals who were present in the second onsite assessment, 505 individuals were excluded due to a lack of electrocardiographic (ECG) information and 368 participants due to missing information on ICVH metrics. Therefore, our main sample consisted of 13,141 individuals. For some sensitivity analyses, the sample was restricted to 12,307 individuals without previous self-reported myocardial infarction, stroke, or heart failure.

### Data collection

ELSA-Brasil protocols^17 , 18^ for anthropometric, clinical and laboratory exams did not change between the first and second assessments. Age, sex, race, education level, and monthly family income were self-reported and stratified accordingly. Monthly income was analyzed as multiples of the Brazilian minimum wage (one Brazilian minimum wage during data collection averaged approximately 310 US dollars). Systolic and diastolic blood pressure (SBP and DBP, respectively) measurements were taken using a validated oscillometric device (Omron HEM 705CPINT). Three measurements were taken at one-minute intervals after a five-minute rest with the subject in the sitting position, and the average of the second and third measurements was considered to define SBP and DBP. Fasting plasma glucose was determined using the hexokinase method and total cholesterol using the cholesterol oxidase method, conducted in an ADVIA 1200 Siemens® equipment.

### AFF diagnosis

ECG recordings in ELSA-Brasil were obtained as described elsewhere.^19 , 20^ Electrocardiography in onsite assessments was performed using a Burdick Atria 6100 device, calibrated at 10 mm/mV and a speed of 25 mm/second. The recordings were transmitted to the reading center in the Minas Gerais Investigation Center. Analyses followed the Glasgow system and were coded according to the Minnesota Coding System. Selected codes (including AFF) were manually reviewed by trained staff. These methods have been adopted in all ELSA-Brasil assessments to date. AFF diagnosis in this article was defined as its presence in ECG tracing at the baseline or on the second visit.

### ICVH score

Detailed information about the ideal ICVH scoring in ELSA-Brasil can be found elsewhere.^21^ The following definitions were used for the seven ICVH metrics, based on the AHA guidelines:^1^

1. Diet: Four adequate components from (a) ≥ 4 servings of fruit and vegetables per day, (b) ≥ 7 ounces of fish per week, (c) ≥ 2 servings of fiber-rich whole grains per day, (d) ≤ 450 kcal of sugar-sweetened beverages per week, and (e) sodium consumption ≤ 1500 mg/day;
2. Physical activity: ≥ 75 minutes/week of vigorous physical activity, or ≥ 150 minutes/week of moderate physical activity, or ≥ 150 minutes/week of moderate + vigorous physical activity;
3. Smoking: never smoked or former smoker age upon quitting of at least two years less than the age at onsite assessment;
4. BMI < 25 kg/m^2^ ;
5. Blood pressure: SBP < 120 mmHg and DBP < 80 mmHg, without antihypertensive medication;
6. Fasting plasma glucose: < 100 mg/dL, without hypoglycemic medication;
7. Total cholesterol: < 200 mg/dl, without lipid-lowering medication.

All information for ICVH scoring was obtained from the second visit, except for the diet ICVH metric. The diet ICVH metric was evaluated using information from the complete food frequency questionnaire, which was applied in the ELSA-Brasil baseline. The global ICVH score was calculated as the sum of the ideal profiles (range: 0 to 7 points). Scores of participants’ lifestyle (diet, physical activity, smoking, and BMI; range 0-4) and health (blood pressure, fasting plasma glucose, and total cholesterol; range 0-3) were evaluated separately.

### Statistical analysis

Categorical variables were presented as counts and proportions, and continuous variables were presented as medians and interquartile ranges. Normality of data was evaluated using density plots and the Anderson-Darling test. We calculated the sensitivity and specificity of different cutoffs for global ICVH score to classify individuals with and without AFF in the sample. We plotted a receiver-operating characteristic (ROC) curve using these values and calculated the area under the ROC curve (AUROC) to estimate the discriminative capacity of the global ICVH scores to identify individuals with AFF in this setting. Binary logistic regression models were built to analyze the association of ICVH metrics and global, lifestyle, and health scores with AFF diagnosis. These models are presented in their crude form and adjusted for age, sex, and race. Sensitivity analyses were performed, restricting the sample to the individuals without previous self-reported myocardial infarction, stroke, or heart failure. After observing a paradoxical association between the total cholesterol ICVH metric and AFF, we ran some analyses *a posteriori,* (1) excluding individuals under statin use, as some authors pointed a potential benefit of statins on incident atrial fibrillation;^22 , 23^ (2) excluding the total cholesterol ICVH metric from the global and health ICVH scores. The crude and adjusted binary logistic regression models were also presented in order to analyze the association between these modified scores and AFF diagnosis. Analyses were performed using R software, version 4.0.0. The significance level was set at 5%.

## Results

Table 1 summarizes the characteristics of the sample. Median age was 55 years and 7,147 (54.4%) were women. Most participants were of white race, had a college education and a family income between six and 15 Brazilian minimum wages (approximately 1,860 to 4,650 US dollars/month). AFF in ECG tracings was detected in 80 (0.6%) participants. The most frequent ICVH metric was smoking, present in 11,548 (87.9%) participants. Supplemental Table 1 shows the characteristics of study participants after excluding those with previous self-reported myocardial infarction, stroke, or heart failure.

**Table 1:** Characteristics of the study sample

	No atrial fibrillation or flutter (N=13,061)	Atrial fibrillation or flutter (N=80)	Total (N=13141)
**Age (years; mean ± SD)**	55.0 [49.0 - 62.0]	64.5 [57.0 - 73.2]	55.0 [49.0 - 62.0]
**Female sex (N (%))**	7117 (54.5%)	30 (37.5%)	7147 (54.4%)
**Race (N (%))**			
White	6750 (52.3%)	46 (60.5%)	6796 (52.3%)
Brown	3625 (28.1%)	21 (27.6%)	3646 (28.1%)
Black	2071 (16.0%)	9 (11.8%)	2080 (16.0%)
Other	471 (3.6%)	0 (0.0%)	471 (3.6%)
**Educational level (N (%))**			
Incomplete high school	1472 (11.3%)	13 (16.2%)	1485 (11.3%)
High school	4110 (31.5%)	25 (31.2%)	4135 (31.5%)
College or above	7476 (57.3%)	42 (52.5%)	7518 (57.2%)
**Monthly family income (N (%))**			
< 6 BMW	2816 (21.7%)	16 (20.0%)	2832 (21.6%)
6 to 15 BMW	6112 (47.0%)	29 (36.2%)	6141 (46.9%)
> 15 BMW	4073 (31.3%)	35 (43.8%)	4108 (31.4%)
**Use of antihypertensive medication (N (%))**	5800 (44.4%)	61 (76.2%)	5861 (44.6%)
**Use of hypoglycemic medication (N (%))**	2577 (20.9%)	30 (39.5%)	2607 (21.0%)
**Use of lipid-lowering medication (N (%))**	6658 (51.0%)	37 (46.2%)	6695 (51.0%)
**ICVH metrics (N (%))**			
Diet	177 (1.4%)	0 (0.0%)	177 (1.3%)
Physical activity	3488 (26.7%)	15 (18.8%)	3503 (26.7%)
Body-mass index	4229 (32.4%)	27 (33.8%)	4256 (32.4%)
Smoking	11474 (87.8%)	74 (92.5%)	11548 (87.9%)
Blood pressure	4499 (34.4%)	7 (8.8%)	4506 (34.3%)
Fasting plasma glucose	5927 (45.4%)	23 (28.7%)	5950 (45.3%)
Total cholesterol	5213 (39.9%)	38 (47.5%)	5251 (40.0%)
**ICVH score (mean ± SD)**			
Global	3.0 [2.0 - 4.0]	2.0 [1.0 - 3.0]	3.0 [2.0 - 4.0]
Lifestyle	1.0 [1.0 - 2.0]	1.0 [1.0 - 2.0]	1.0 [1.0 - 2.0]
Health	1.0 [0.0 - 2.0]	1.0 [0.0 - 1.0]	1.0 [0.0 - 2.0]

*BMW: Brazilian minimum wage; one Brazilian minimum wage during data collection averaged approximately 310 US dollars. ICVH: Ideal cardiovascular health. Source: The authors.*

Supplemental Figure 1 shows the ROC curve addressing the accuracy of global ICVH scores in identifying individuals with AFF in the sample. The AUROC is 0.59, indicating a relatively low discriminatory power of global ICVH scores in this scenario. More details are shown in the Supplemental Table 2 , which displays sensitivity and specificity values for different global ICVH score cutoffs.

**Table 2:** Odds ratios (95% CI) for the association between ICVH scores and metrics and atrial fibrillation or flutter diagnosis

	All sample		Excluding patients with self-reported myocardial infarction, stroke or heart failure	
	Crude	Adjusted for age, sex and race	Crude	Adjusted for age, sex and race
**Global ICVH score**	0.80 (0.67 - 0.95)*	0.96 (0.80 - 1.16)	0.87 (0.71 - 1.08)	1.01 (0.81 - 1.26)
**Lifestyle ICVH score**	0.95 (0.71 - 1.26)	0.96 (0.70 - 1.30)	1.00 (0.70 - 1.42)	0.96 (0.67 - 1.38)
Diet	^†^	^†^	^†^	^†^
Physical activity	0.63 (0.36 - 1.11)	0.65 (0.37 - 1.15)	0.71 (0.36 - 1.38)	0.67 (0.34 - 1.31)
Body mass index	1.06 (0.67 - 1.69)	1.19 (0.74 - 1.92)	1.33 (0.77 - 2.32)	1.38 (0.79 - 2.40)
Smoking	1.71 (0.74 - 3.93)	1.40 (0.60 - 3.23)	1.08 (0.46 - 2.52)	0.92 (0.39 - 2.18)
**Health ICVH score**	0.66 (0.51 - 0.85)*	0.96 (0.73 - 1.26)	0.77 (0.57 - 1.03)	1.06 (0.77 - 1.46)
Blood pressure	0.18 (0.08 - 0.40)*	0.33 (0.15 - 0.74)*	0.23 (0.10 - 0.53)*	0.36 (0.15 - 0.87)*
Glucose	0.49 (0.30 - 0.79)*	0.79 (0.48 - 1.31)	0.65 (0.37 - 1.13)	0.97 (0.54 - 1.74)
Total cholesterol	1.36 (0.88 - 2.12)	1.88 (1.19 - 2.98)*	1.62 (0.95 - 2.79)	2.14 (1.23 - 3.72)*

ICVH: Ideal cardiovascular health. † Odds ratio estimates for the association between the diet ICVH metric and atrial fibrillation or flutter could not be obtained due to the lack of individuals with an optimal profile of diet and a diagnosis of atrial fibrillation or flutter; * p<0.05. Source: The authors.

Table 2 shows the odds ratios (OR) and respective 95% confidence intervals (95%CI) for the association of ICVH scores and metrics with prevalent AFF. In the entire sample, ICVH scores were inversely associated with AFF diagnosis in crude models (p=0.012), although this association disappeared vanished after adjustment for age, sex, and race (p=0.70). No lifestyle ICVH metric nor the lifestyle ICVH score was significantly associated with AFF. The blood pressure ICVH metric was inversely associated with AFF in both crude (p<0.001) and adjusted (p=0.007) models. There was a paradoxical positive association between the total cholesterol ICVH profile and AFF in the adjusted models (p=0.007). The exclusion of individuals under statin use from analyses led to a loss of significance for the association between the total cholesterol ICVH profile and AFF in the adjusted models (OR: 2.22; 95%CI: 0.86 - 5.70; p=0.098). However, this loss of significance seems to be primarily due to a lack of statistical power, as 46.2% of participants with AFF and 51.0% of participants without AFF were under statins.

As the associations of AFF with blood pressure and total cholesterol ICVH metrics were antagonistic, AFF was not associated with an overall health ICVH score in adjusted models (p=0.76). After excluding individuals with self-reported myocardial infarction, stroke, or heart failure, the associations between AFF and blood pressure (p=0.023) and total cholesterol ICVH metrics (p=0.007) in adjusted models remained.

We also verified whether the global and/or health ICVH scores were associated with AFF after the exclusion of the total cholesterol ICVH metric. The global ICVH score was shown to be inversely associated with an AFF diagnosis in crude models (OR: 0.70; 95% CI: 0.57 – 0.86; p=0.001) with a non-significant trend towards an inverse association in adjusted models (OR: 0.84; 95% CI: 0.67 – 1.04; p=0.11). Health ICVH scores were inversely associated with an AFF diagnosis in both crude (OR: 0.41; 95% CI: 0.28 – 0.59; p<0.001) and adjusted (OR: 0.63; 95% CI: 0.42 – 0.93; p=0.020) models.

## Discussion

In the ELSA-Brasil sample, global ICVH scores were associated with prevalent AFF in crude models. However, they were associated with low AUROC values and statistical significance disappeared in models adjusted for age, sex, and race. Analyzing ICVH metrics separately, a strong inverse association was found between the blood pressure ICVH metric and AFF. A positive but paradoxical association was observed between the total cholesterol ICVH metric and AFF: an ideal total cholesterol ICVH metric was associated with AFF diagnosis. The restriction of the sample to individuals with no self-reported myocardial infarction, stroke, or heart failure yielded similar results. The exclusion of the total cholesterol ICVH metric led to a significant inverse association between health ICVH scores and AFF.

The importance of hypertension as a risk factor for AFF is well defined in the literature. In a systematic review of risk factors for incident AFF, Allan et al.^24^ reviewed data from 32 population-based cohorts, including more than 20 million subjects. The authors found that the diagnosis of hypertension and high systolic blood pressure were consistently associated with incident AFF. Recently, Rattani et al.^25^ used data from the ARIC study to calculate the population attributable risk of hypertension for AFF. The authors defined hypertension according to the criteria from the 7th Report of the Joint National Committee (≥140/90 mmHg) and the 2017 American Heart Association/American College of Cardiology guidelines (≥130/80 mmHg), and found population attributed fraction values of 11% and 13%, respectively. Expectedly, the presence of an ideal blood pressure profile was also inversely associated with AFF in the ARIC,^10^ REGARDS,^11^ and MESA^9^ cohort studies.

Although total cholesterol (mainly due to its main subfraction, LDL-c) is a major risk factor for atherosclerotic cardiovascular disease, its association with AFF is much less clear. Other authors have pointed to the cholesterol paradox in AFF, describing lower cholesterol levels in individuals with AFF, upon comparing controls in diverse scenarios, such as individuals treated in referral centers for other cardiovascular diseases, those treated in primary care units,^26 , 27^ or even those from community samples.^28^ In a recent systematic review, Guan et al.^29^ found that high levels of total cholesterol (defined at cutoffs between 220 and 260 mg/dL across studies, or based on the empirical distribution within the sample) were associated with a pooled hazard ratio for AFF of 0.81 (95% CI: 0.72-0.92), which was consistent with our findings. In the same systematic review, analyses using LDL-C instead of total cholesterol levels yielded similar results.

There is some evidence that statins may have a potential benefit on incident atrial fibrillation.^22 , 23^ However, the ICVH metric considers as a non-ideal profile if the individual is under lipid-lowering medications, regardless of his/her total cholesterol levels. Therefore, it could be argued that the paradoxical association between total cholesterol ICVH profile and AFF is explained by statin use. Our data suggest this is not the case. The proportion of individuals in our sample using lipid-lowering medication is similar. Moreover, although the exclusion of individuals under statins led to a loss of statistical significance, this was probably due to the high number of exclusions from this sensitivity analysis (51.0% of our sample), as the OR point estimates were also similar (1.88 for the whole sample; 2.22 after excluding those using lipid-lowering medication)

The criterion used as the total cholesterol ICVH metric (<200 mg/dL without lipid-lowering medication use) is stricter than those in the studies included in this systematic review. The cohort studies^9 - 11^ that evaluated ICVH scores and their relationship with AFF diagnosis have also studied the total cholesterol ICVH metric separately. In all cases, the hazard point estimates suggested higher odds for AFF in individuals with ideal total cholesterol, but without statistical significance. Their non-significant results contrast to our finding of a significant, positive, but paradoxical association between the total cholesterol ICVH metric and AFF diagnosis in adjusted models. This significant association in our study was evident even with less AFF cases in the sample, when compared to these previous publications. Differences in study populations and design may be partly responsible for this discrepancy. Similar to the United States, black Brazilians have a higher prevalence of hypertension^30^ and a lower prevalence of dyslipidemia,^31^ when compared to white Brazilians. It could be argued, given the strong inverse association between blood pressure and AFF, that race could be a confounder in the positive association between a total cholesterol ICVH profile and AFF diagnosis. However, it is important to note that our results were maintained even after adjustment for race. Future longitudinal analyses from the ELSA-Brasil, including the occurrence of more AFF cases, will clarify if the cholesterol paradox in individuals with AFF is particularly strong in the Brazilian population.

In 2018, Isakadze et al.^32^ proposed that the use of the ICVH score to improve overall cardiovascular health may substantially reduce AFF prevalence. Although current evidence suggests this is probably true, our results and previous data also point out that individual ICVH metrics may have different (and even antagonistic) effects on AFF prevalence. Therefore, it is arguable that the progress in AFF prevention resulting from improvements in ICVH scores must be estimated considering not only the impact of all ICVH metrics together but also of each metric separately.

The present study has strengths. First, it evaluated data from a large sample in Brazil. To our knowledge, this is the first study to analyze the association between AFF and ICVH metrics in a South American sample with different characteristics compared to the samples analyzed in previous studies. ECG tracings were also analyzed in a centralized reading center, using a standardized protocol,^19^ and the ICVH score criteria could be applied with minimal adaptations.

This study also needs to be interpreted in the context of its limitations. The sample of this study included a small number of AFF participants. This is most probably due to its high proportion of individuals of less than 60 years of age. As time progresses, incident AFF cases may increase the power of our analyses. This may have influenced our finding of non-association between AFF diagnosis and ICVH scores. The food frequency questionnaire was not applied in the second visit of patients in the ELSA-Brasil; therefore, it was considered that participants maintained their diet ICVH status after the baseline. The prevalence of an ICVH diet was very low in the ELSA-Brasil baseline (1.3%),^21^ which is consistent with the findings from other populations.^33^ Therefore, it is unlikely that a large proportion of participants would have adopted an ideal diet between the baseline and the second onsite assessment. The AFF was defined based only on ECG tracings of the ELSA-Brasil and, although this ensures high specificity, paroxysmal AFF may be underrepresented in our analyses.

## Conclusions

In conclusion, no significant associations were observed between global ICVH scores and AFF diagnosis in our analyses, at least partially due to antagonistic associations of AFF with blood pressure and total cholesterol. Our results suggest that estimating the AFF prevention effect using global ICVH scores may not be adequate, and ICVH metrics should be considered in separate.

## * Supplemental Materials

For additional information Supplemental Figure 1, please click here .

For additional information Supplemental Tables, please click here .
